# Unravelling the veil of appearance anxiety: exploring social media use among Chinese young people

**DOI:** 10.1186/s40359-023-01495-7

**Published:** 2024-01-02

**Authors:** Yihan Wu, Ying Xue, Xiaohan Zhao, Sijia Han, Weiyun Wu

**Affiliations:** School of Social Development, Nanjing Normal University, Nanjing, China

**Keywords:** Chinese, Young people, Grounded theory, Appearance anxiety, Social media use

## Abstract

The purpose of this study is to explore the relationship between appearance anxiety and social media use among Chinese adolescents. Using a grounded theory approach, the study conducted two-round online interviews with ten Chinese university students and subsequently constructed a theoretical model of social media appearance anxiety among Chinese young people. The results of the study indicate that social media has a dual impact on appearance anxiety. On one hand, increased social media engagement amplifies appearance anxiety by shaping aesthetic standards and fostering comparative environments. On the other hand, diverse aesthetic perspectives and authentic presentations on social media partially alleviate appearance anxiety by promoting acceptance of unique appearances and boosting self-confidence. It is emphasized in this study that there should be an in-depth understanding of the dual impact and complicated relationship of social media on the daily lives of Chinese adolescents to further develop relevant strategies that promote healthy social media behavior among youth. Furthermore, this study calls for efforts to actively promote the healthy image and psychological well-being of adolescents while alleviating the negative impact of appearance anxiety and overall mental health. Such efforts are needed to ensure a positive and healthy development for the younger generation.

## Introduction

As digital natives, young people depend heavily on social media and use a variety of platforms to meet both their practical needs and spiritual requirements. Against the backdrop of normalized epidemic prevention and control measures for COVID-19, numerous universities in China with local outbreaks must implement closed management and offer online teaching, while some students require medical observation at designated locations. These measures increase the amount of time that college students spend on social media, exacerbating their reliance on it and possibly affecting their psychological well-being [1]. Therefore, investigating the relationship between social media use and appearance anxiety among young people is crucial to help correctly recognize the issue of appearance anxiety and understand the mechanisms through which social media use impacts appearance anxiety positively or negatively.

Social media use refers to the utilization of virtual tools or platforms on the internet for social interaction with others [1, 2]. In modern times, a variety of different social media have become popular means for individuals to engage in social communication and establish social connections, including social networking sites or platforms, instant messaging applications, blogging sites, and online virtual gaming programs.

Appearance anxiety is a form of social evaluative anxiety prevalent in the era of influencer culture [3]. It specifically pertains to an individual’s preoccupation with their physical appearance and the possibility of negative social evaluations based on it, leading to persistent negative emotions such as worry, distress, fear, and dissatisfaction [3–5]. This phenomenon encompasses not only specific physical features related to one’s appearance, such as skin color, nose shape, eye shape, and face shape, but also body image characteristics associated with one’s outward appearance, such as height, weight, and muscle proportion [6]. Despite scholars’ lack of consistent, standardized definition of “appearance anxiety”, this study defines it as a negative emotion that causes individuals to experience anxiety regarding their external appearance, which is broader than just a medical condition.

Recent research on appearance anxiety and social media use primarily focuses on the negative effects of specific social media platforms on users’ appearance anxiety [1]. Steinsbekk et al. [2] demonstrated that other-oriented social media use can reduce adolescents’ self-esteem regarding their appearance, indicating the potential impact of social media usage purposes on appearance anxiety. Additionally, problematic usage behaviors or addiction may also be associated with individuals’ experience of appearance anxiety. Caner et al. [3] conducted a web-based survey of Turkish high school students and found that social media usage time, the influence of social media influencer’s sharing, and close attention to online gaming were all significant factors leading to appearance anxiety. While these quantitative studies have shown some negative effects of certain motivation and behavioral factors of social media use on appearance anxiety, they lack exploration of interactive relationships between different factors and mechanisms underlying them.

From an individual self-perspective, scholars argue that engaging in more activities related to appearance or body correlates with an increased likelihood of developing psychological problems related to appearance. Jarman et al. [7] conducted a large-scale survey of Australian adolescents using the Body Image Social-Cultural Attitudes Scale and found that high levels of attention to appearance-related social media use were significantly negatively correlated with body satisfaction and happiness. When individuals engage in more online activities related to selfies, receiving more negative feedback from others could lead to greater appearance anxiety [8]. Seeking social approval during the process of building online intimate relationships is also a crucial factor contributing to appearance anxiety [3, 4]. Moreover, social comparison has a mediating effect on appearance anxiety [9]. In studies on body image, Lonergan et al. [10] demonstrated that attention to and editing and sharing of selfies could lead to more negative body image and increased body dissatisfaction. Fadouly et al. [11] also demonstrated that negative social comparison could result in unpleasant experiences, with users who frequently use social media platforms having more concerns about their bodies and daily diet than other users, leading to more negative emotions. While these studies on body image partially complement the limited perspective of appearance anxiety research on the impact of body dissatisfaction, they lack sufficient attention to other important influencing factors of appearance anxiety and features of social media use that contribute to appearance anxiety.

In the field of media studies, some scholars have used qualitative analysis to deeply describe the functional role of social media and its impact on the psychological health of adolescents. For example, Zhang and Chen [12] explored the cognitive judgments and attitudes or behavioral patterns of female adolescents when they first encountered the concept of appearance anxiety, revealing that the media’s communication of appearance anxiety is built on a normal level of dissatisfaction with self-cognition and a pursuit of icing on the cake. Liu [13] used grounded theory to reveal the shaping role of mass media on the commodification of women’s appearance and individual aesthetic views, as well as their impact on women’s appearance anxiety. These studies partially compensate for the shortcomings of the social macro perspective in social media usage research and emphasize the important impact of social media’s own information dissemination and shaping on appearance anxiety among young women.

Recent research from different perspectives has explored the potential positive effects of social media use on appearance anxiety. Tiggemann and Zinoviev [14] demonstrated through quantitative research that sharing and posting real photos without filters can prevent users from feeling appearance concerns. This may be because such self-disclosure satisfies adolescents’ needs for interpersonal relationships and emotional expression, promoting their subjective sense of happiness [9]. Hjetland et al. [15], through focus group interviews with 27 adolescents from two high schools in Norway, proposed that media use as a stage for peer interaction can help adolescents feel social support, which is worth considering for their psychological health. Similarly, due to the impact of the COVID-19 epidemic, more and more domestic and foreign scholars have paid attention to mental health problems caused by the epidemic. These studies consistently demonstrate that online chatting can alleviate anxiety, depression, and other mental health problems caused by restrictions on face-to-face communication, as well as reduce risky behaviors [16, 17], and suggest that more use of social media can meet people’s social communication needs and promote their psychological health [18]. It is worth noting that the existing research focuses mainly on exploring the promotion of mental health by social media use under the background of the epidemic, with almost no research attempting to explore the potential positive effects of social media use on appearance anxiety from different motivations for social media use. On the other hand, based on existing research evidence [7–9], the impact of social media on appearance anxiety may lead individuals to engage more in activities related to appearance or self-presentation due to the impact of the epidemic, increasing their focus on their appearance and triggering psychological changes related to appearance. So far, only Cristel et al. [19] found through a simple online survey that the increased use of video conferencing during the epidemic has led users to pay more attention to appearance in videos and suggested that this may trigger dissatisfaction with appearance and choice of cosmetic options based on a highly appearance-oriented perspective.

Although previous research has made significant contributions to the study of the impact of social media on appearance anxiety, identifying a notable correlation between social media and appearance anxiety, as well as potential influencing factors, there are still several limitations that need to be addressed. Firstly, current research primarily focuses on young people when examining appearance anxiety related to social media. While young people are the predominant users of social media, there is a lack of comprehensive information regarding their characteristics and descriptions of appearance anxiety caused by social media use. Secondly, existing studies have not fully explored the various objective external factors that contribute to appearance anxiety, emphasizing the need to consider individual differences and comprehensively investigate the influence of social media on appearance anxiety. Furthermore, appearance anxiety stemming from social media is a multidimensional and intricate issue that necessitates further exploration and analysis. Lastly, existing research solely relies on quantitative survey methods, failing to capture the dynamic mechanisms and interactions between social media use and appearance anxiety.

Accordingly, this study employed grounded theory to attain a more comprehensive understanding of appearance anxiety among Chinese young people and its relationship with social media. Grounded theory facilitates a thorough comprehension of the phenomenon under investigation [20], thereby enabling a deep exploration of the impact and underlying mechanisms of social media on appearance anxiety. By employing grounded theory, the perspectives and experiences of the research participants are considered, resulting in a more profound understanding. Moreover, grounded theory allows for the development of a fresh theoretical framework that elucidates how social media influences appearance anxiety. This framework not only addresses existing research gaps but also provides a basis for future investigations. The dynamic mechanisms between social media use and appearance anxiety can be effectively discovered through grounded theory, hence it is a suitable methodology for the current study.

## Methods

The current study started in September 2022 and ended in June 2023. Before the official interviews in October 2022, 3 preliminary interviews were conducted with young people. The feedback from these interviews was used to modify and improve the interview outline. The study consisted of two formal interviews, based on relevant literature and the preliminary interviews. The first interview focused on gathering participants’ basic information and introducing the concepts, while the second interview explored the experiences and factors related to young people’s appearance anxiety caused by social media.

Researchers issued a research invitation letter on popular Chinese social media platforms such as Weibo and Zhihu in November 2022. The invitation letter clearly outlined the research objectives, duration of interviews, and the purpose of the study The researchers utilized a theoretical sampling method to select participants who aligned with the study’s objectives based on specific selection criteria. These criteria included demographic factors such as age (i.e., college-aged students) and country (Mainland China), as well as relevant experiences related to appearance anxiety. In order to ensure a diverse sample and gain deeper insights into appearance anxiety among young people, participants from various academic majors and genders were selected to ensure greater representation.

Following participant’s consent, 10 eligible individuals were selected for 2 round in-depth interviews to achieve theoretical saturation. Throughout the interview process, researchers reiterated to the interviewees that the interviews and audio recordings were solely intended for research purposes. Any personal information related to the interviewees would be kept strictly confidential. Furthermore, the interviewees retained the right to refuse answering any questions or terminate the interview if they so desired. The basic information of the interviewees is provided in Table 1.

The interviews were conducted with a duration ranging from 1 to 2 h. Efforts were made to extend the interview content based on individual variances and in-depth exploration of participants’ experiences regarding appearance anxiety influenced by social media, thereby acquiring valuable textual information. All interviews were conducted online via internet platforms. The entire interview process was recorded and subsequently transcribed into a Word document. This Word document served as the basis for data analysis and coding.

**Table 1:** Participants’ background information

No.	Gender	Age	Major	Grade
P1	Male	18	Law	Year 1
P2	Female	20	Applied Psychology	Year 1
P3	Male	20	Physical Geography and Natural Environment	Year 3
P4	Female	20	Bioengineering	Year 3
P5	Female	20	Sociology	Year 3
P6	Female	21	Sociology	Year 3
P7	Male	21	Chinese Language and Literature	Year 3
P8	Male	21	Mechanical Design and Manufacturing and Automation	Year 3
P9	Male	21	Computer Science and Technology	Year 3
P10	Female	21	Social Work	Year 4

This study adhered to the foundational principles of grounded theory, employing three distinct modes of coding for the original textual data. Firstly, building upon a preliminary analysis of the interview material, the researcher identified various open codes. Secondly, through iterative categorization, organization, and differentiation of the open codes, the authors synthesized them into 10 axial codes, including time of using social media, motivation of using social media, method of using social media, relieves appearance anxiety, motivation for self-improvement, a platform for self-presentation, satisfying psychological needs such as social interaction, amplifies appearance anxiety, shaping a biased aesthetic and setting a uniform aesthetic standard, losing oneself between ideal and reality.

After conducting a comprehensive examination of the interview data, and carefully analyzing and discriminating the open and axial codes, the research team identified four selective themes. To further enhance the credibility and rigor of the analysis, the codes were reviewed and validated by two additional researchers who were involved in the study. This process of data triangulation and investigator triangulation ensured that multiple perspectives were considered, thereby enhancing the overall trustworthiness and reliability of the study’s findings.

## Results

The four themes developed through grounded theory are, appearance anxiety under self and peer evaluation, characteristics of social media use of Chinese young people, the negative influence of using social media on appearance anxiety, and the positive influence of using social media on appearance anxiety.

### Theme 1: Appearance anxiety under self and peer evaluation

All the young people in the study agree that they experience different levels of appearance anxiety. This anxiety is caused by various external factors such as complexion, facial shape, physique, height, facial features, hairline, skin color, and teeth. They feel dissatisfied with their appearance, which is the main reason for their anxiety. For example

My appearance is very ordinary, and even has some noticeably unattractive characteristics. For example, my facial expression appears chubby, and my posture is not good, which is largely influenced by my lifestyle habits. Personally, I believe I have some appearance anxiety, as it is impossible to completely disregard the opinions of others in modern society. (20 years old, female, P4).

If perfection is rated as a ten, I would probably give myself a five for my appearance. Sometimes, I do experience a bit of appearance anxiety, because there are so many attractive people around and when you compare yourself to them, anxiety tends to creep in without realizing it. (21 years old, female, P6).

In addition to their own dissatisfactions with their appearance, many young people express concerns about how their appearance may put them at a disadvantage in romantic relationships, interviews for job applications and further study. For example:

On the other hand, I believe that for many students, if they want to pursue romantic relationships, they must possess slightly better physical attributes. I think this could also be a source of anxiety. (20 years old, male, P3).

I think that if you stand next to someone who is more attractive and has an aggressive personality, you will be at a disadvantage. In the future, this could affect your job and academic interview scores. (20 years old, female, P2).

In today’s society, appearance has become a “*bonus point*” in the realms of romance and job interviews. Based on the interviews from participants of this study, it is evident that they believe the importance of “*appearance*” has increased within today’s social evaluation system. This phenomenon of emphasizing “*appearance*” is closely related to the prevalent “*judging people by their appearance*” trend in the market-oriented society of today. From the perspective of young people, their desire to “improve their appearance” and the confusion surrounding how to fulfill their desire further exacerbate their appearance anxiety. For example:

I don’t know how to make adjustments, so I haven’t made any. Because I don’t know how to change myself, I feel even more anxious. (21 years old, male, P9).

On the other hand, anxiety may stem from the desire to improve oneself, as one also wishes to appear more attractive. (20 years old, male, P3).

Therefore, from “s*elf-image anxiety*” to “*preoccupation with attractiveness*” and finally to the urgent yet bewildered desire to “*look better*”, appearance anxiety is universally those young people, generated and developed within their social environment.

Furthermore, the evaluations and attitudes of others serve as a reflective “*mirror*” influencing their perception and assessment of their own appearance. It is worth noting that discrepancies exist not only between self-evaluations and evaluations from others but also among evaluations from different individuals. When there are disparities between self-evaluation and evaluations from others, those young people tend to be more inclined to accept the assessments of others. On the other hand, when there are differences in evaluations from different individuals, young people are more likely to place greater importance on the evaluations of individuals who hold significant cognitive value to them, particularly romantic partners. These tendencies serve as both the causes and manifestations of appearance anxiety among young people.

I find myself without a clear conception of my own appearance. If someone thinks I look good, it makes me happy; if someone thinks otherwise, it brings about feelings of unhappiness. Additionally, my appearance anxiety is easily influenced by the opinions of my romantic partner. For example, if my current boyfriend thinks I am attractive, it fills me with joy; however, if my next boyfriend perceives me as unattractive, it instills a profound sense of insecurity within me. (21 years old, female, P10).

The evaluation of my own appearance primarily stems from the evaluations of others. (20 years old, male, P3).

When there are discrepancies between my own evaluation of my appearance and the evaluations from others, I am more inclined to believe the assessments of others, lacking confidence in my own evaluation. I believe this, too, constitutes a form of appearance anxiety. (21 years old, male, P7).

Thus, it is evident that others play a significant role in the formation and development of appearance anxiety among university students. Despite acknowledging these tendencies as a form of appearance anxiety, young people are unable to escape from its grasp.

### Theme 2: Characteristics of social media use of Chinese young people

After young people enter university, the overall time of social media use increases due to easier coursework compared to high school and more freedom on campuses. Some of the most frequent applications used by these young people are bilibili, QQ, WeChat (Moments), Weibo, Zhihu, Little Redbook, TikTok. Among them, social media platforms which focus on sharing pictures and videos, such as TikTok, Little Redbook, and WeChat (Moments) would generate more anxiety about appearance

Since I started engaging with platforms like TikTok and Little Redbook, my anxiety has intensified. For instance, there are some extraordinarily attractive girls on TikTok, and I inevitably find myself comparing my own appearance to theirs. Moreover, my ex-boyfriend was also fond of scrolling through videos of beautiful women on TikTok, which only fueled my self-comparisons. As a consequence, I can’t help but feel inadequate. It seems that TikTok is specifically designed to inundate you with likes and comments for these stunningly gorgeous women, leaving you with the impression that everyone on the platform is more attractive than you. (21 years old, female, P10).

Some people always send the WeChat (Moment), their photos are taken quite good. I do not take pictures, and rarely take pictures, it does not look good. (21 years old, male, P8).

In these social platforms full of pictures and videos, the attention of young people is captivated by the visual manifestations of individuals blessed with remarkable aesthetic appeal. This visual eminence engenders an unspoken realm of comparison wherein individuals inadvertently develop an implicit sense of inadequacy regarding their own physical appearance, ultimately leading to the emergence of facial insecurities These covert comparisons oftentimes fall within the purview of upward social comparison. Social media has veritably burgeoned as the preeminent conduit for such societal juxtapositions. As young people peruse pictures and videos on platforms which focus on “*life-sharing*”, they are inadvertently immersed in a milieu of comparison through the vicarious sharing of photos and videos that serve as embodiments of self-presentation.

### Theme 3: The negative impact of social media use

Owing to the copious influx of information, the expeditious dissemination, and the interactive nature of social media platforms, a myriad of channels emerges facilitating comparisons between young individuals and the purveyors of online information. Social media, with its pervasive reach, exerts a magnifying influence on the anxieties surrounding one’s physical appearance among the youth. While certain dimensions of social media consumption afford aesthetic gratification to these impressionable minds, they simultaneously engender an atmosphere that fosters self-comparisons and engrossment in perceived blemishes of their own visage:

When I brush some videos in Jitterbug and B station, the platform will recommend some good-looking girls or boys. I feel that my body and face are much different from theirs. It makes me feel more anxious and not so good, and I don’t want to see these anymore. Sometimes I will quickly brush past or search for other content after seeing it, so that the big data can brush it off and push other content. However, sometimes my friends will share this with me, and I can’t help it. If he shares, I will definitely click on it to take a look, polite response, because Shake Yin you will have a ‘read’ after reading, so he saw that I have seen the video he shared, not so embarrassing. The second is to push the acne treatment and other such ads to make their faces look better, I watch those ads, I will have the urgent feeling of ‘want to make their own acne also no’, very anxious, anxious. Also, the skin care commercials always show a face full of pimples, which makes me think of myself and make me feel uncomfortable. (18 years old, male, P1).

Secondly, social media plays a significant role in influencing and even shaping the aesthetics of those young people and “*setting*” the evaluation standards for their appearance:

The standards on social media are often much higher than in real life. Because social media is full of carefully selected photos, and because you can speak freely online, many people can be very critical and even offensive. For example, if some ordinary-looking people post photos in Zhihu, they are likely to be attacked for being ugly and so on. In this way, social media spreads appearance anxiety. I sometimes brush up on some of the requirements for a relationship partner, such as how tall you have to be, and if I don’t meet that standard, I’ll care. It’s this social stereotypical standard or something like that. I will also go to appreciate some good-looking male or female celebrities, this is still often there: because I often brush those clips on the B site: the world’s top ten beautiful men, Chinese ancient beautiful men ……. I’m still quite concerned, will look at other people’s evaluation of their face; or how he went on Zhihu to evaluate a person’s body, looks and other things. The influence of these on me is still quite large, because this look more, will certainly have an impact on your entire aesthetic. (20 years old, male, P3).

In addition, the functions of retouching software and special effects on social media make young people obtain the ideal image, but the gap between the constructed ideal image and the real image makes them feel disappointed and even more confused about the orientation of “*self*”, for example:

All I post on WeChat are nice personal photos. I will take them only after I put on makeup, and I will upload them only after I fix them. Every time I send it out, I will be complimented by others, and I will feel very happy to be complimented, but not necessarily if I’m a vegetarian. After removing the makeup and reading their comments, I felt that the photo was so fake. Because my photos look better on the internet, no one will say that there are people in reality who have negative comments about my looks. I know that the me presented on the internet is a more ideal me created by myself, not the me in real life. But in order for others to see a better me to be recognized, so I will choose to P-picture, but I know in my heart that I don’t look like this. (21 years old, female, P10).

Observably, in this contemporary era of digitization, social media has erected a realm of communication for these young people, proffering an abundant array of veritable or transcendent cultural artifacts that bear symbolic import. These young people, through the conduits of social media, acquire copious amounts of information, integrating the discussed and appraised contents into their own cognition and tangible existence. Their aesthetic faculties and assessments of physical appearance become subtly influenced by the insidious impact of the “*standards*” set forth by iconic visages exhibited on social media platforms. However, once the gaze of these young minds, entranced by the meticulously curated facades presented on social networks, shifts upon the visages encountered in their real-life spheres, an inherent disappointment ensues, readily engendering the psychological phenomenon known as “*facial anxiety*”.

When I compare my photos with those of my thin and fat days, I think the former looks better and I can buy clothes better. It is mainly a comparison with my classmates. When I see others who are thin, long and straight, I feel good, regular and healthy, and being fat is also a reflection of undiscipline, poor body management and slovenliness. This point of view is certainly problematic, but combined with my own specific situation, I feel that I really do not eat much as a child, what task to grasp to complete, to maintain good learning that state. But after I put on weight, people are getting lazy, gluttonous and procrastinating. I don’t know if it’s something biologically influenced or what, I should say it’s my own experience, and being triggered by information I see on social media, and not everyone is like that. But I think most people, if they keep the habit of self-discipline, his body is usually not more bloated, but the lean, dry type (20 years old, female, P2).

Each social culture presents its own distinct “*ideal body*”, which is propagated through social media. Consequently, the culture and societal norms exert a positive influence on physical attributes that align with such values, while casting a negative light upon those that deviate from them. For example, female figures frequently portrayed on social media tend to embody allure and slender physiques, traits that align with the socially advocated ideals of femininity. These images are often associated with positive attributes, such as “*being good, disciplined, and healthy*”. Conversely, images that deviate from this idealized physical appearance are frequently linked with negative connotations and undesirable characteristics, such as being overweight, undisciplined, or unkempt. Within this context, young people find themselves comparing their own bodies to the repeatedly emphasized “*ideal body*” dictated by society and any disparity between their own physique and the perceived ideal can give rise to obstacles in body imagery, leading to feelings of insecurity, diminished self-satisfaction, and disrupted eating patterns.

### Theme 4: The positive effects of social media use

However, despite being aware of the amplified role of social media in exacerbating appearance anxiety, why do young people still indulge in its use? Why do they continue to internalize and adhere to the “*stereotypical standards*” prevalent on social media, even while being ensnared in the grip of numerous appearance-related anxieties? Delving into the key reasons, this study found that these young people, under the latent premise of acknowledging and willingly enduring the risk of being embroiled in the propagation of appearance anxiety through social media, are driven by their pursuit of the “*ideal self*” social interaction and self-presentation, entertainment and leisure, as well as the need for information, all of which can be fulfilled through social media. For instance, although social media fosters a climate of societal comparison and preoccupation with appearance, it also provides them with avenues for alleviating their anxieties:

There is another blogger who looks more like me and has a similar face shape. I think people tend to be attracted to people who resemble them. I aspire to her attitude, her life. I previously thought my square face shape was very unattractive, but after I looked at her, I felt she was just beautiful and felt it eased my looks anxiety. I feel like there are two different voices on social media right now. Most of the time it’s the plastic surgery face that is more in line with popular aesthetics, but after such a long time, people around me are getting more and more awake and starting to like healthy looks. Although the former is dominant, I feel that the latter is becoming more and more powerful, for example, by observing the development of my favorite bloggers you can also see this. (20 years old, female, P2).

I will watch some makeup. But I can’t learn if I watch it, or I don’t learn it seriously. Some bloggers have a big difference between before and after makeup, which makes me feel that I really look better after makeup. I feel that it will relieve some of my anxiety about my appearance because some bloggers like to make a big difference between before and after, which makes me feel that it’s okay to look average and that I can look good with makeup. (21 years old, female, P10).

Some classes will ask for a camera, some won’t. I don’t mind too much. There are times when I don’t wake up and I don’t look good with the camera on. Since everyone looks the same in the video conference, and no one looks particularly good, it relieves my anxiety about my appearance a little. (21 years old, male, P8).

Henceforth, it becomes evident that social media offers more than just upward comparison, it is not perpetually characterized by idealized visual depictions far removed from reality. Furthermore, diverse aesthetic perspectives emerge and gain prominence. Such revelations engender a greater inclination among young people to embrace their own appearances, temporarily liberating themselves from the pursuit of an unattainable ideal image.

During the interview, while the prevailing viewpoint still acknowledges the predominantly adverse impact of appearance anxiety, there are also instances where young people perceive it as a catalyst for positive change in their personal aesthetics:

I look every ten minutes, half an hour to see if anyone has commented or something. Good comments and likes will be my motivation to work hard and my self-confidence will be strengthened so that I can make more efforts to post such photos again and try to achieve this effect without beauty someday. (21 years old, male, P8).

I am concerned about cosmetics, dressing and photo poses, because I hope to find the right way to improve my appearance through these external ways, as recommended by different bloggers. (21 years old, female, P6).

It becomes apparent that social media enables young people to enhance their appearance and outward image by learning from individuals with superior physical attractiveness. It also grants them access to positive evaluations, bolstering their self-confidence and providing impetus for self-improvement.

In addition, although there are differences in the frequency of posting among young people, their motives can be summarized into three points: recording, sharing and communication. Regardless of the motivation, today, communication between social media is an important part of their socialization, and young people have a positive, expectant, and valued mentality toward interactions on social media. As a result, more people are posting content involving personal photos on social platforms where there are more acquaintances. This is because the feedback posted on these social media platforms with more acquaintances basically has two characteristics, namely, the feedback is less about comments on looks, and even if the comments are related to looks, they are less offensive, more positive and more friendly in language. This adds to the positive emotional experience for young people.

I will share my life on social media and share fun, significant things in my circle of friends. In this way, I would find people who also care about these things to connect with. I want to find some friends that I can connect with and relate to through my circle of friends, and also let others know how I am doing. (21 years old, male, P7).

Posting updates on one hand is to record, and on the other hand is to share and interact. I see other people’s comments as an interactive process, and I think it is a very interesting process, which is considered a positive influence? If there are no comments, I think I would be more lost, because I would like to know what others think, and I also have expectations for interaction. (20 years old, female, P5).

### The proposed model

**Fig. 1:**
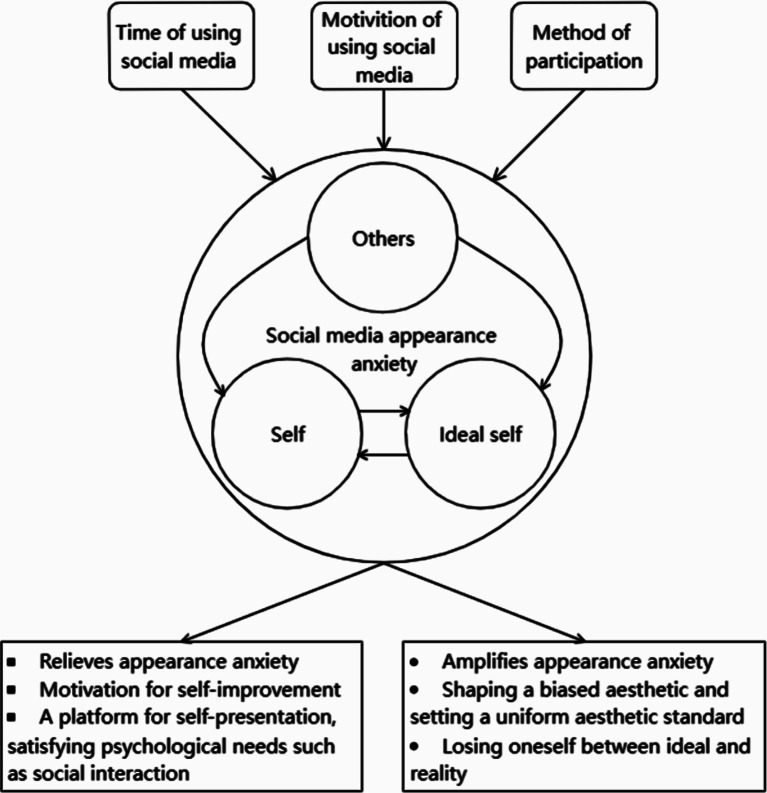
Constructed theoretical model of social media appearance anxiety among Chinese young people

The proposed model of “social media appearance anxiety among Chinese young people”, was illustrated in Fig. 1, visually represents the interactions between codes and their impact on young people’s appearance anxiety.

As shown in Fig. 1, the theme of “appearance anxiety under self and peer evaluation” highlights how young people’s dissatisfaction with their own appearance and the opinions of others can contribute to appearance anxiety. The evaluations and attitudes of peers and society act as a mirror, influencing how young people perceive their own appearance. This theme establishes the foundation for understanding the link between appearance anxiety and social media use. The theme of “characteristics of social media use of Chinese young people” describes how young people extensively use photo and video-centric social media platforms to document and share their daily lives. These platforms create an environment where young people can compare themselves to others. Visual-oriented social media platforms immerse adolescents in a comparative environment, which can intensify appearance anxiety as they strive to conform to societal beauty standards.

The theme of the “negative influence of using social media on appearance anxiety” explains how social media platforms contribute to appearance anxiety. The constant influx of information and interactivity facilitates comparisons between young people, fueling appearance anxiety. Additionally, social media platforms often frame and amplify societal beauty standards, shaping young people’s values and expectations. The use of filters and editing features further widens the gap between idealized images and reality, leading to disappointment and self-doubt. The theme of the “positive influence of using social media on appearance anxiety” acknowledges that social media can also have positive effects on appearance anxiety. Diverse beauty standards and authentic representations on social media can promote positive internal and external improvements. Moreover, the use of social media allows young people to build social networks, increase positive emotional experiences, and fulfill their social needs.

These four themes interact with each other to impact appearance anxiety. Yong people’s dissatisfaction with their own appearance and the opinions of others (theme 1) are reinforced by the comparative environment and societal beauty standards found on social media (theme 2). The constant exposure to idealized images and comparisons on social media platforms (theme 3) can contribute to heightened appearance anxiety. However, social media also has the potential to provide a platform for diverse beauty standards, authentic representations, and positive experiences (theme 4), which can counteract appearance anxiety to some extent.

In summary, the interplay between these four themes helps explain how social media’s impact on appearance anxiety is influenced by self and peer evaluation, characteristics of social media use, both negative and positive effects of social media, and the complex interactions between these factors.

## Discussion and conclusion

The present study unveils that the use of social media among Chinese young people constitutes a double-edged sword in terms of their appearance anxiety. On the one hand, as young people enter university, the intensity of their social media use increases, and the platforms amplify their appearance anxiety by shaping aesthetic standards and creating a comparative field. On the other hand, the diverse aesthetic perspectives, authentic portrayals, and positive evaluations found on social media partly alleviate appearance anxiety among these young people.

The influence of social media in shaping aesthetic standards and its impact on appearance anxiety has been consistently affirmed by numerous existing studies [3, 7, 9, 11, 13, 16, 21–23]. However, it is noteworthy that the existing studies about appearance anxiety remains focused on young females, this study adds the perspective of some young males about appearance anxiety, emphasizing the prevalence of social media in amplifying appearance anxiety. Based on the interviews with the participants, both female and male students often compared their “*ideal self*”, which were embellished by means of retouching and other means on social media, with reality. This comparison creates a gap between the ideal and the reality, leading to disappointment and negative feelings about themselves, which in turn leads to appearance anxiety. However, males’ appearance anxiety issues tend to be more hidden and underappreciated on social media than females. This may be because traditional societal expectations of men focus on other aspects, such as career success and financial independence, rather than physical appearance. As a result, males’ concerns and anxieties about their appearance tend to be overlooked. Thus, this study is groundbreaking in that it expands the understanding of appearance anxiety from being limited to women to the male population. It reveals the pervasive and obvious impact of social media on both males and females, suggesting that we need to focus on and address this issue more comprehensively. Understanding and recognizing the stress and anxiety men face in social media can help promote a healthier and more inclusive social media environment that provides a positive physical and mental health experience for all.

A large number of studies have addressed the negative impact of monolithic aesthetic standards on appearance anxiety in social media [12, 13, 24], and this study further found that since there is not just an upward comparison on social media, there is not just an ideal body image that presents a far cry from reality. Due to the diversity of aesthetic perspectives that have emerged in recent years on social media, and social media provides a platform that allows users to be exposed to and identify with multiple aesthetic perspectives and body images. By showcasing diversity and positive comments, social media has gone some way to alleviating young people’s appearance anxiety. For example, consider the rise of body-positive influencers on social media platforms. These influencers share their personal stories, promote self-love, and challenge societal beauty standards. They encourage their followers to embrace their bodies and focus on overall well-being rather than conforming to a specific appearance ideal. Such influencers create supportive online communities where individuals can openly discuss mental health and body image struggles. These online communities focused on self-care, body positivity, and mental well-being provide support and understanding for those facing appearance anxiety. Users can share their experiences, seek advice, and receive validation, creating a sense of belonging and reducing feelings of isolation. The study found that social media provided users with the opportunity to express themselves and embrace diversity, thereby alleviating young people’s over-reliance on traditional aesthetic standards and the body dissatisfaction and anxiety associated with them. This finding further confirms the dual role of social media on appearance anxiety. Although social media still has the negative effect of shaping aesthetic standards and triggering comparison behaviors, it also presents users with positive possibilities as a major way to positively alleviate young people’s appearance anxiety [14]. In addition, the main forum for young people to post developments (especially those involving personal photos) lies in acquaintance social platforms, which has a positive effect on protecting them from appearance anxiety.

In conclusion, based on the results of this study, our study suggests that young people should clarify the causes of personal appearance anxiety, remove themselves from the “*information cocoon*” created by social media, experience and feel the real world, improve their cognitive and self-control abilities, and further strengthen their self-identity. For example, young people can choose to follow some positive accounts and avoid following too much appearance-themed content to avoid increased self-comparison and anxiety. It is also a good idea to periodically disconnect from social media to give yourself some space away from the phone and the Internet to focus on real life and self-growth. For universities, they can rely on campus cultural activities through counseling and classroom education to enhance the aesthetic sensibilities and enrich the spiritual world of young people. It is important to note that social media is not entirely negative; it also provides many opportunities for communication, learning and connection. Therefore, while paying attention to social media, education should cultivate good media literacy and educate students to learn to distinguish the authenticity of information and maintain rational thinking and multicultural values.

Nevertheless, there are some limitations in this study. For example, the data collection time period of this study included exactly the point in time when China’s epidemic control policy changed; therefore, the epidemic policy and the changes and potential impacts brought about before and after the epidemic were not taken into account. Therefore, the potential impact of these factors on young people’s appearance anxiety needs to be taken into account in future studies. Such a study could provide a more comprehensive understanding of the impact of social media on appearance anxiety in different settings and provide more accurate guidance for the development of relevant interventions and support measures.

## Data Availability

The data and materials that support the findings of this study are available from the authors upon reasonable request.
